# Supplementary material for: Rosin-enabled ultraclean and damage-free transfer of graphene for large-area flexible organic light-emitting diodes
Source: Nat Commun. 2017 Feb 24;8:14560. doi: 10.1038/ncomms14560 (PMC5333113; doi:10.1038/ncomms14560)
Supplement: Supplementary Information — Supplementary Figures, Supplementary Tables, Supplementary Notes and Supplementary References [file ncomms14560-s1.pdf]

Supplementary Note 1: OLEDs and OPV cells with different graphene TCEs

Supplementary Table 1 and 2 summarize the device structure, performance, and active area of organic light-emitting diodes (OLEDs) and organic photovoltaic (OPV) cells with different graphene transparent conductive electrodes (TCEs) reported in the literature so far. It can be seen that most of the OLED devices with graphene TCEs have a lighting area much less than 0.1 cm^2. Only a few devices can reach about 1 cm^2 in lighting area. For OPV cells with graphene TCEs, the active areas are only 0.0004 to 0.6 cm^2. In our work, a 4-inch flexible monolithic OLED device with a uniform lighting area of 56 cm^2 and high brightness of about 10,000 cd m^{-2} has been fabricated on an 8×9 cm^2 PET substrate for the first time by using a rosin-transferred 5-layer graphene film as anode. In addition, our OLEDs with a lighting area of 0.16 cm^2 (typical size for the reported graphene-based OLEDs) show a high maximum current efficiency (CE) and power efficiency (PE) of 89.7 cd A^{-1} and 102.6 lm W^{-1}, respectively, which are comparable to the best values of the graphene-based OLEDs reported in the literature without any light-coupling structures and cavity resonance enhancement design. Moreover, our graphene anode is very stable. In contrast, the reported OLEDs with a comparable performance usually use graphene films doped by HNO_3 or $AuCl_3$ as the anode, and these are very unstable and can greatly degrade device efficiency and lifetime. These results show the superiority of our ultraclean and damage-free graphene TCEs fabricated by the rosin-supported transfer method for large-area high-performance flexible OLEDs.

Supplementary Table 1. OLEDs made with different graphene (G)-TCEs.

Ref.	G-TCEs	R_s (Ω per square)/ T @ 550 nm	Device structure	Maximum CE (cd A^{-1})	Maximum PE (lm W^{-1})	Lighting area (cm^2)	Emission color
1	Multilayer (anode, ~20 L)	310/~85%@522 nm	Al/Glass/G/V_2O_5/NPB/CBP:Ir(ppy)$_2$(acac)/Bphen/Bphen:Cs_2CO_3/Sm/Au	~ 0.75	~ 0.38	_	Green
2	Monolayer (anode)	700-800/96.6%	PET/G/MoO_3/NPB:MoO_3/TAPC/CBP:Ir(ppy)$_2$(acac)/TPBi/Liq/Al	~ 11.44	~ 2.24	0.09	Green
3	Monolayer (anode, doped by TiO_x and PEDOT:PSS)	86/94.1%	Glass/G-TiO_X-PEDOT:PSS/NPB/Alq$_3$:C545T/Alq$_3$/LiF/Al	10.11 @ ~ 1000 cd m^{-2}	5.41 @ ~ 1000 cd m^{-2}	0.1	Green
4	Bilayer (anode)	~ 754.2/~95%	Al/Glass/G/V_2O_5/NPB/Alq$_3$/Bphen:Cs_2CO_3/Sm/Au	1.18	0.41	0.09	Green
5	SDBS-Graphene composite electrode (GCE, anode)	80±10/79%	PET/GCE/PEDOT:PSS/TPD/Alq$_3$/LiF/Al	3.9	_	0.1	Green
6	Reduced graphene oxide (rGO, anode)	~ 800/~82%	Quartz/rGO/PEDOT:PSS/NPD/Alq$_3$/LiF/Al	_	~ 0.35	~ 0.00785	Green
7[a]	Multilayer (4L, anode, prepared by layer-by-layer transfer but PMMA or thermal release tape was used	54/~90%	PET/G/GraHIL(PEDOT:PSS+PFI)/NPB/Bebq2:C545T/Bebq2/Liq/Al	30.2	37.2	~ 0.06	Green
			PET/G/GraHIL(PEDOT:PSS+PFI)/TAPC/TCTA:Ir(ppy)$_3$/CBP:Ir(ppy)$_3$/TPBi/LiF/Al	98.1	102.7	~ 0.06	Green

	only in the first transfer step, therefore there are no polymer residue particle between each layer, doped by HNO_3)		PET/G/GraHIL(PEDOT:PSS+PFI)/NPB/TBADN:NPB:rubrene/TBADN:NPB:DPAVBi/TBADN:DPAVBi/$Bebq_2$/BaF_2/Al	16.3	_	~ 0.06	White
8	Monolayer (anode)	1500±200/96.4%	Glass/G/PEDOT:PSS(AI4083)/Phenyl substituted PPV/ZnO NPs and Ionic Solution {(PEO+$TBABF_4$) in acetonitrile}/Al	0.18	_	~ 0.06	Yellow
	Monolayer/PEDOT:PSS hybrid (anode)	~ 90±10/92.8%	Glass/G/PEDOT:PSS(PH1000)/PEDOT:PSS(AI4083)/Phenyl substituted PPV/ZnO NPs and Ionic Solution {(PEO+$TBABF_4$) in acetonitrile}/Al	0.89	_	~ 0.06	Yellow
9	Multilayer (5~8L, anode)	<200/80-85%	Glass/G/HTL/EL/ETL/LiF/Al	_	~ 3	0.04	Blue
10	Multilayer (4L, anode, doped by CYTOP)	200/>85%	Glass/CYTOP-G/NPB:WO_3/Alq_3:C545T/Alq_3/LiF/Al	_	_	0.0025	Green
11	Multilayer (4L, anode)	50/~90%	Glass/G/PEDOT:PSS/NPB/Alq_3/LiF/Al	3.3	_	_	Green
12	rGO-Multilayer (anode, doped by HNO_3)	>300/~65%	Glass or Quartz/rGO/PEDOT:PSS(AI4083)/NPB/Alq_3/Liq/Al	4.5	_	0.09	Green

13	Graphene-CNT hybrid films (anode, SWCNT/PBASE/FLG(bilayer)/PBASE/FLG(bilayer), modified by PBASE	76/~89.13%	Quartz/G-CNT/PEDOT:PSS with PFI/NPB/CBP:Ir(ppy)$_3$/BAlq$_3$/Alq$_3$/LiF/Al	~ 14.7	9.2	_	Green
14	Multilayer (12L for anode, Au NP doping; 8L for cathode, Ag NW doping)	~ 110/~80% for anode; ~741/~80% for cathode	Quartz/G/PEDOT:PSS/PVK/CdZnSeS/ZnS/ZnO NP/G	~ 0.45	_	_	Green
15	Reduced chemically derived graphene (rCDG)	~5000/-	Quartz/rCDG/SY+PEO+KCF$_3$SO$_3$/Ca/Al	1.5	_	0.05	Yellow
			Quartz/rCDG/KCF$_3$SO$_3$+PEO+SY/PEDOT:PSS	9	5	0.05	Yellow
16	Multilayer (4 L, Cathode, doped by CsF)	118/84.9% @ 500 nm	Glass/ITO/HAT-CN/VB-FNPD/PVK:Firpic/PFN:CsF/G:CsF	3.1	_	0.06	Blue
17	Monolayer (anode, doped by MoO$_3$)	~ 590/94%	Glass/G/MoO$_3$/CBP:MoO$_3$/CBP/CBP:Ir(ppy)$_3$/TPBi:Ir(ppy)$_3$/TPBi/LiF/Al	55 @ 1000 cd m^{-2}	32 @ 1000 cd m^{-2}	_	Green
	Multilayer (3 L, anode, doped by MoO$_3$)	~ 70/-	Glass/G/MoO$_3$/CBP:MoO$_3$/CBP/CBP:Ir(ppy)$_2$(acac)/TPBi/Liq/Al	65 @ 1000 cd m^{-2}	34 @ 1000 cd m^{-2}	_	Green
18	Cu/graphene composite (anode)	~ 0.0039/-	Cu/G/V$_2$O$_5$/NPB/Alq$_3$/Alq$_3$:C545T/Bphen:Cs$_2$CO$_3$/Sm/Au	6.1	7.6	0.04	Green

19	Multilayer graphene (5 - 8L, anode, O_2 plasma treated)	552/>90%	Glass/G/TAPC/HAT-CN/TAPC/HAT-CN/TAPC/TCTA:FIrpic/DCzPPy:FIrpic/BmPyPB/LiF/Al	_	24.1 @ 1000 cd m^{-2}	0.04	Blue
20	Monolayer (anode, doped by V_2O_5)	~610/>90%	Glass/G/V_2O_5/CBP:MoO_3/CBP/CBP:Ir(ppy)$_2$(acac)/TPBi/Liq/Al	_	~ 85 @ 1000 cd m^{-2}	_	Green
	Monolayer (anode, doped by WO_3)	~780/>90%	Glass/G/WO_3/CBP:MoO_3/CBP/CBP:Ir(ppy)$_2$(acac)/TPBi/Liq/Al	_	~ 65 @ 1000 cd m^{-2}	_	Green
21	Monolayer G/Ag/AZO (anode)	-/~77%	PET/G/Ag/AZO/NPB/Alq_3/LiF/Al	1.46	_	0.24	Green
22[b]	Monolayer (anode, doped by Triethyloxonium hexachloroantimonate (OA))	<200/~97%	Plastic or Glass/G/MoO_3/PEDOT:PSS/CBP:MoO_3/CBP/CBP:Ir(ppy)$_2$(acac)/TPBi/LiF/Al	~ 245 @ 1000 cd m^{-2}	~ 200 @ 1000 cd m^{-2}	_	Green
		<200/~97%	Plastic or Glass/G/MoO_3/PEDOT/CBP:MoO_3/CBPCBP:Ir(ppy)$_2$(acac):Ir(MDQ)$_2$(acac)/CBP:Ir(ppy)$_2$(acac)/CBP:Firpic/TPBi/TPBi/LiF/Al	~ 130 @ 1000 cd m^{-2}	~ 90 @ 1000 cd m^{-2}	_	White
23	3L graphene with the top layer being selectively oxidized (G/GO anode)	263/90.7%	PET/G/GO/MoO_3/TAPC/Ir(ppy)$_2$(acac):TCTA/Ir(ppy)$_2$(acac):Bphen/Bphen/Li/Al	82.0	98.2	0.16	Green

24	4L graphene (anode, doped by TFMS)	63.3/88.3%	PET/G/DNTPD/TAPC/TCTA:Ir(ppy)$_2$(acac)/CBP:Ir(ppy)$_2$(acac)/TPBI/LiF/Al	104.1	80.7	1	Green
25[c]	4L graphene High-index TiO_2 layers were placed underneath graphene (anode, doped by HNO_3)	92.5± 9.4/90% (Prepared by quadruple repetition of growth of an SLG on a copper foil and subsequent wet-transfer, doped by HNO_3)	Glass/TiO_2/G/GraHIL/TAPC/TCTA:Ir(ppy)$_2$acac/CBP:Ir(ppy)$_2$acac/TPBi/LiF/Al	168.4 (257.0 with a half-ball lens attached)	160.3 (250.4 with a half-ball lens attached)	_	Green
			Glass/TiO_2/G/GraHIL/TAPC/TCTA:Ir(ppy)$_2$acac/CBP:Ir(ppy)$_2$acac/TPBi/BCP:Li/MoO_3/TAPC/TCTA:Ir(ppy)$_2$acac/CBP:Ir(ppy)$_2$acac/TPBi/LiF/Al	_	120.8 (183.5 with a half-ball lens coupled)	_	
		330.3± 16.1/88% (Direct growth of MLG on a Ni layer coated on a SiO_2/Si wafer, followed by wet transfer, doped by HNO_3)	PET TiO_2/G/PEDOT:PSS/ MoO_3 /CBP /1:1 co-host of CBP: B3PYMPM doped with 7% Ir(ppy)2acac /B3PYMPM /LiF/Al	_	155.8	_	
26[d]	3L or 4L graphene sandwiched between TiO_2 and WO_3 (anode, doped by CYTOP)	470-80/97.3% -82.5% for 1-5L	Glass/TiO_2/G/WO_3/CBP:WO_3/CBP:tris(1-phenylisoquinolinato-C2,N)iridium(III)/BCP/LiF/Al	_	26	0.0025	Green
This work	3L G/GO (anode)	180/91.4%	PET/G/GO/MoO_3/TAPC/Ir(ppy)$_2$(acac):TCTA/Ir(ppy	89.7	102.6	0.16	Green

			$)_2$(acac):Bphen/ Bphen/Li/Al			
	5L G/GO (anode)	120/85.1%	The same as above	_	_	56

[a] A flexible white OLED was fabricated on a 5 cm × 5 cm PET substrate. Roughness estimated from the picture, the lighting area is around 2 cm × 2 cm although it was not clearly defined.

[b] To enhance the efficiency of OLEDs, light-coupling methods including substrates and lenses were used.

[c] High-index TiO_2 layers were placed underneath the graphene to enable cavity resonance enhancement.

[d] TiO_2/graphene/WO_3 electrodes can enhance the microcavity resonance, thereby increasing the power efficiency.

Supplementary Table 2. OPV cells made with different graphene TCEs.

Ref.	G-TCEs	Rs (Ω per square)/T@550 nm)	Device structure	Active area (cm^2)	PCE (%)
27	CVD few-layer graphene	210-1350/ 72-91%	Glass/G/PEDOT:PSS/P3HT:PCBM/LiF/Al	_	0.21
28	Multilayer graphene (3L)	300/91.2%	Quartz/G/PEDOT:PSS/CuPc/C_{60}/BCP/Ag(or Mg/Ag)	0.0121	1.63
29	Reduced graphene oxide (rGO)-CNT hybrid	~ 600/87%	Glass/rGO-CNT/PEDOT:PSS/P3HT:PCBM/ Ca:Al	0.04	0.85
30	Multilayer graphene (4L), doped by HCl and HNO_3	~ 80/~90%	Quartz/G/MoO_3/PEDOT:PSS/P3HT:PCBM/LiF/Al	0.04	2.5
31	rGO	17900/69%	Quartz/rGO/PEDOT:PSS/P3HT:PCBM/LiF/ Al	_	0.13
32	rGO	3200/65%	PET/rGO/PEDOT:PSS/P3HT:PCBM/ TiO_2/Al	_	0.78
33	rGO	1000/80%	Quartz/rGO/PEDOT:PSS/P3HT:PCBM/Al	0.0004	1.01± 0.05%
34	rGO	1600/70%	PET/rGO/PEDOT:PSS/P3HT:PCBM/Al	0.18	1.1
35	CVD graphene	230/72%	PET/G/PEDOT:PSS/CuPc/C_{60}/BCP/Al	0.0075	1.18
36	CVD multilayer graphene	520-850/85-90 %@ 450nm	Glass/G/WPF-6-oxy-F/P3HT:PCBM/PEDOT :PSS/Al	0.0466	1.23

	cathode				
37	AgNW-graphene hybrid cathode	34.4±1.5/92.8%	Glass/G-AgNW/ZnO/P3HT:PCBM/MoO_3/Ag	_	3.3
38	Multilayer graphene (10L)	-/-	Glass/ITO/ZnO/P3HT:PCBM/GO/G	0.1	2.5
39	CVD multilayer graphene as interlayer	500~700/-	Glass/ITO PEDOT:PSS/P3HT:PCBM/MoO_3/G/MoO_3/ZnPc:C60/LiF/Al	0.04	2.9
40	Multilayer graphene (3L)	300±12/ 91.8±0.4%	Quartz/G/D-HIL(PEDOT:PEG(PC)-PEDOT:PSS)/DBP/C_{60}/BCP/Al	0.0121	2.9
41	Graphene/Ag	83000/47%	PET/G/Ag/PEDOT:PSS/P3HT:C_{60}/Al	0.6	0.18
42	rGO micromesh (rGOMM)	565/59%	PET/rGOMM/PEDOT:PSS/PCDTBT:$PC_{71}BM$/TiO_x/Al	0.04/1.35	3.05/1.07

Supplementary Note 2: Comparison of the basic properties of rosin with other polymers used for graphene transfer

Supplementary Table 3 shows the basic physical and chemical properties of rosin, commonly-used PMMA and other organic small molecule polymers used for graphene transfer. It can be seen that natural organic small molecule rosin has a low molecular weight (*ca.* 302), a low adsorption energy ($E_{ad.}$) of 1.04 eV with a graphene film, and super solubility in organic solvents including alcohol, ether, benzene and chloroform, *etc*. The low $E_{ad.}$ is beneficial for the separation of the rosin layer from the graphene surface. Good solubility allows the rosin to be easily dissolved in the commonly used chemical solvents. Therefore, the rosin can be removed very easily from the graphene surface by solution washing without damaging the graphene structure, leading to ultraclean and damage-free transfer.

Supplementary Table 3. Basic properties of rosin and other polymers used for graphene transfer.

Ref.	Polymer	Molecular weight	Molecular structure	Molecular formula	$E_{ad.}$ (eV)	Natural (Yes/No)	Solubility in organic solvents
3, 43, 44	PMMA	500000~ 1000000	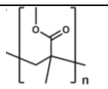	$(C_5H_8O_2)n$	>>1.45[a]	No	Slight
45	Pentacene	278.35	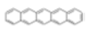	$C_{22}H_{14}$	1.45	No	Slight
2	SPPO1	517	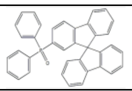	$C_{37}H_{25}OP$	0.69	No	Good
This work	Rosin	302	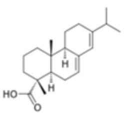	$C_{20}H_{30}O_2$	1.04	Yes	Good

[a] 1.45 is the adsorption energy of HMMA ($C_{31}H_{52}O_{12}$, a very short chain of PMMA polymer for reducing computation time), which should be much lower than that of PMMA due to its much higher molecular weight and longer chain.

Supplementary Note 3: Theoretical calculations of $E_{ad.}$ of different polymers on graphene

For graphene transfer, an ideal polymer support layer should have a weak interaction with graphene, which is beneficial for its separation from the graphene surface. Here we calculated $E_{ad.}$ values for different polymers with graphene with density functional theory (DFT), including rosin with major components of resin acids (primarily abietic acid), 2-(diphenylphosphory) spirofluorene (SPPO1, $C_{37}H_{25}OP$), pentacene ($C_{22}H_{14}$), and HMMA ($C_{31}H_{52}O_{12}$, a very short chain of PMMA polymer in order to reduce computation time).

The $E_{ad.}$ of rosin for the most stable configuration on a graphene surface was calculated to be 1.04 eV (Fig. 1a in the main text). In contrast, the $E_{ad.}$ of HMMA for

the most stable configuration on graphene is 1.45 eV (Fig. 1b), which is about 1.4 times higher than that of rosin. Furthermore, it is worth noting that the average molecular weight of PMMA is hundreds of thousands to millions, much higher than that of HMMA (616). Therefore, the $E_{ad.}$ of PMMA on a graphene surface should be much larger than that of HMMA. Combined with the low solubility of PMMA in organic solvents, these explain why the commonly-used PMMA-supported transfer always leads to the presence of severe PMMA residue and damage to the graphene.

In 2015, Kim *et al.* reported the use of pentacene, a polycyclic aromatic hydrocarbon, as a support layer for graphene transfer[45]. The $E_{ad.}$ of pentacene with graphene was reported to be 1.95 eV in ref. 45, while it was calculated to be 1.45 eV in our work (Fig. 1c). The different calculated E_{ad}. may result from the different generalized gradient approximation functional of Perdew-Burke-Ernzerh type that was used during the theoretical calculations. In addition, similar to PMMA, pentacene has a low solubility in organic solvents. Therefore, pentacene is also difficult to completely remove after transfer, as shown in Fig. 2 in ref. 45.

In 2014, Han *et al.* reported that a layer of SPPO1 could be inserted between PMMA and graphene to achieve the efficient transfer of graphene[2]. Our DFT calculations indicate that the SPPO1 molecule prefers to take a stable adsorption configuration on the graphene surface as shown in Fig. 1d with an $E_{ad.}$ of 0.69 eV, which is much lower than that of PMMA and pentacene. However, the SPPO1 layer cannot be used alone since it is too brittle to retain the integrity of the graphene film during the solution transfer process[2]. Although the PMMA layer can help to keep the

integrity of graphene film during transfer, it cannot be completely removed after transfer because of its low solubility in organic solvents. As a result, some large PMMA residue particles can be observed on graphene in AFM images [2].

Supplementary Note 4: Rosin-enabled transfer of large-area graphene films by a substrate etching method

Supplementary Fig. 1 shows a schematic of the transfer process of a large-area CVD-grown graphene film from a Cu foil to a target substrate using rosin as the support layer. Supplementary Fig. 2 shows a typical SEM image of CVD-grown graphene on a Cu substrate and an OM image of the transferred rosin/graphene stack on a SiO_2/Si substrate. Graphene islands and wrinkles can be clearly observed through the semi-transparent rosin layer. However, no micro-cracks can be found in the rosin layer, indicating that it is strong enough to support the graphene film during the transfer process. This is significantly different from the SPPO1 supporting layer reported in Supplementary ref. 2.

It can be seen from Supplementary Fig. 5 and Fig. 2 in the main text that the rosin-transferred graphene films are very clean and free of damage. Only a few tiny rosin residue particles (less than 5 nm) were occasionally observed in HRTEM images. Even the islands, graphene edges and wrinkles with a high absorption ability are free of rosin residue. As a result, they show a very low R_{max} (the maximum height of residue particles) up to 15 nm (Supplementary Fig. 6).

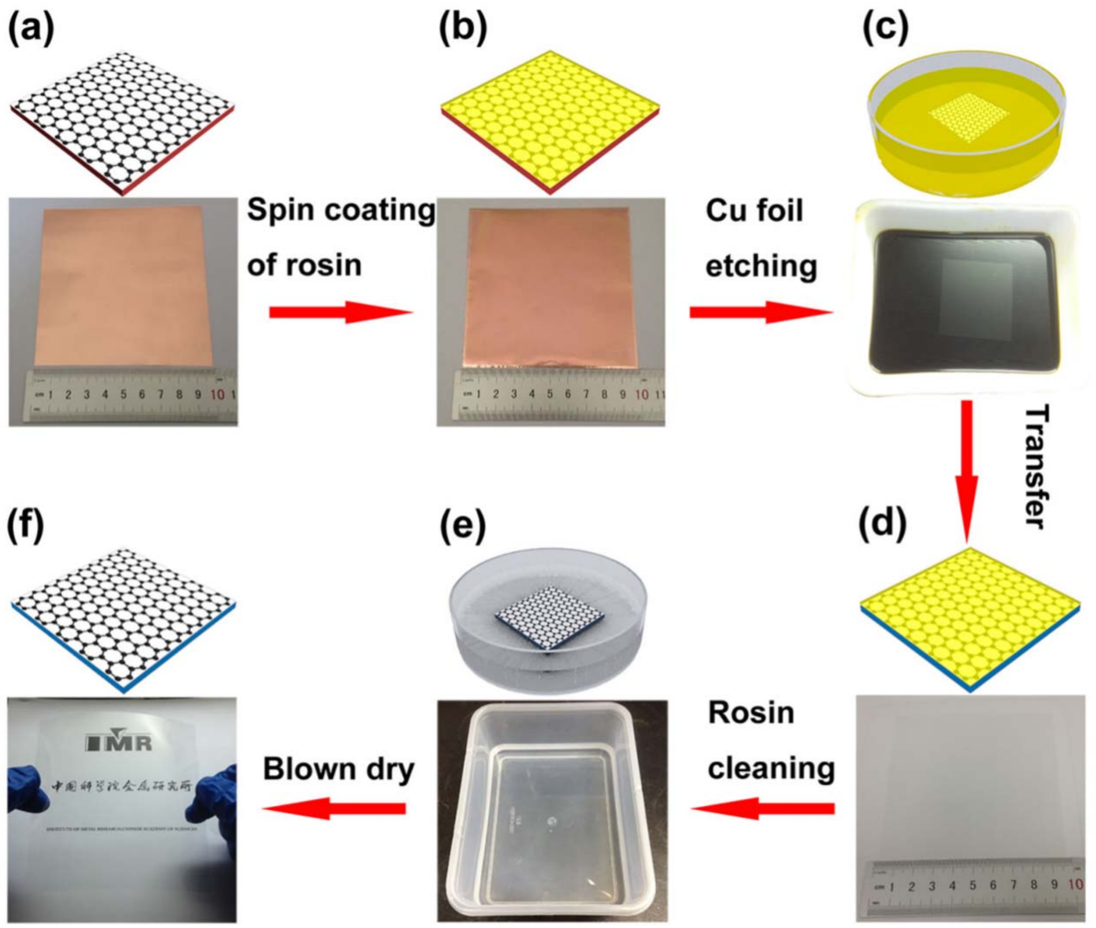

Supplementary Figure 1. Schematic of the transfer process of a large-area CVD-grown graphene film from a Cu foil to a target substrate (PET, Quartz, SiO_2/Si) using a rosin film as the support layer. The bottom pictures show the transfer of graphene onto PET. (a) A CVD-grown graphene film on a Cu foil, (b) a rosin/graphene/Cu stack obtained by spin coating a thin layer of rosin, (c) a floating rosin/graphene stack after removing the Cu foil by $FeCl_3$ etching, (d) a rosin/graphene/target substrate stack obtained by collecting the floating rosin/graphene stack with the target substrate, (e) a graphene/target substrate stack in an organic solvent for the removal of the rosin layer, and (f) monolayer graphene on a target substrate, which has been blow-dried using high-purity nitrogen.

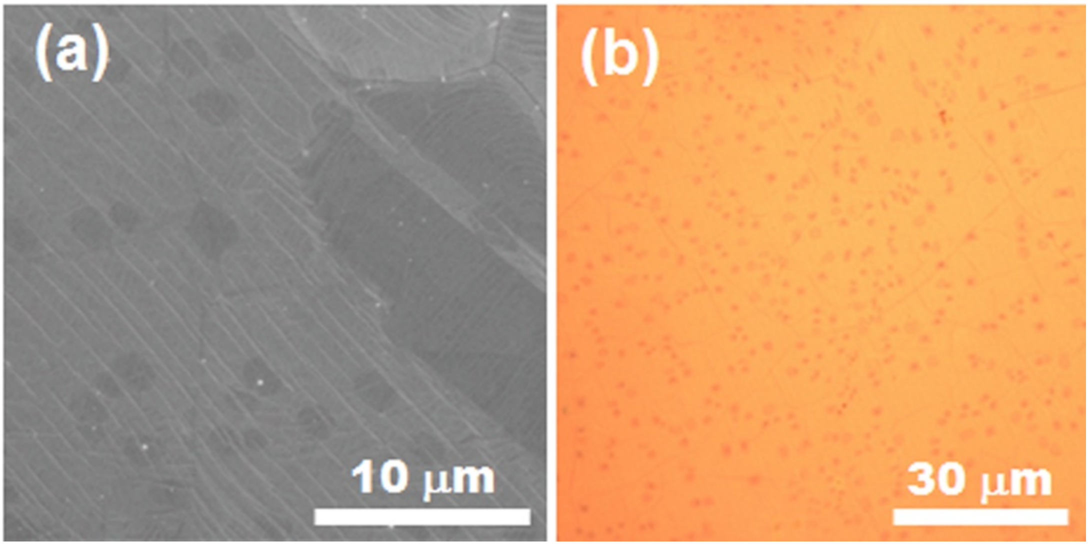

Supplementary Figure 2. **Surface structure characterization.** (a) SEM image of CVD-grown graphene on a Cu foil, (b) typical OM image of a rosin/graphene stack transferred onto a SiO_2/Si substrate.

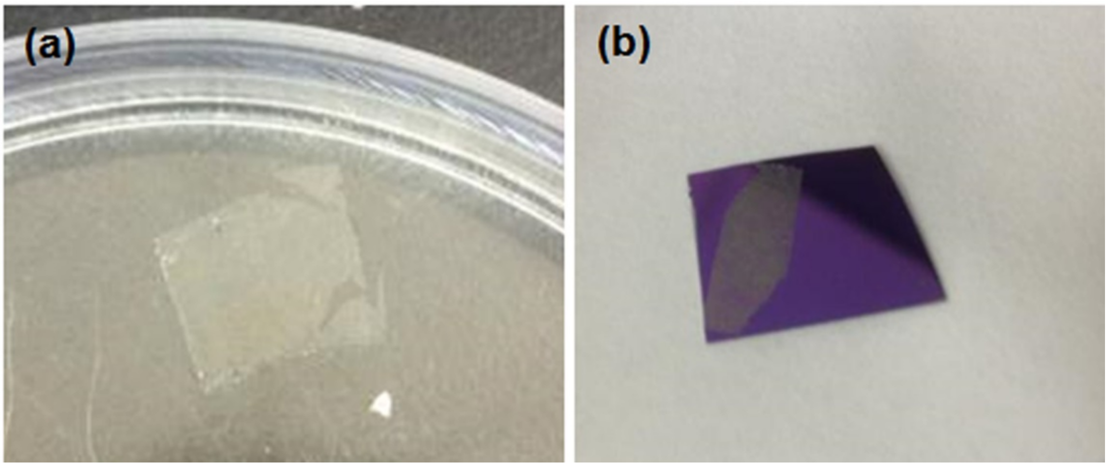

Supplementary Figure 3. **Transfer of a CVD-grown graphene film (1 cm^2) from a Cu foil to a SiO_2/Si substrate using a spin-coated rosin film (20 wt% rosin in ethyl lactate)**. (a) A floating rosin/graphene stack in DI water after removing the Cu foil by $FeCl_3$ etching, showing obvious tearing. (b) A rosin/graphene stack collected on SiO_2/Si substrate, showing obvious damages of the transferred graphene film.

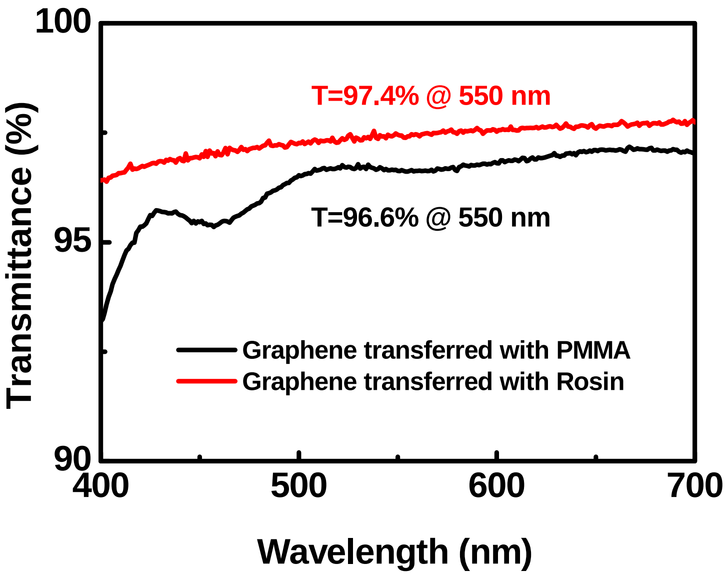

Supplementary Figure 4. The transmittance spectra of rosin- and PMMA-transferred graphene films on a PET substrate.

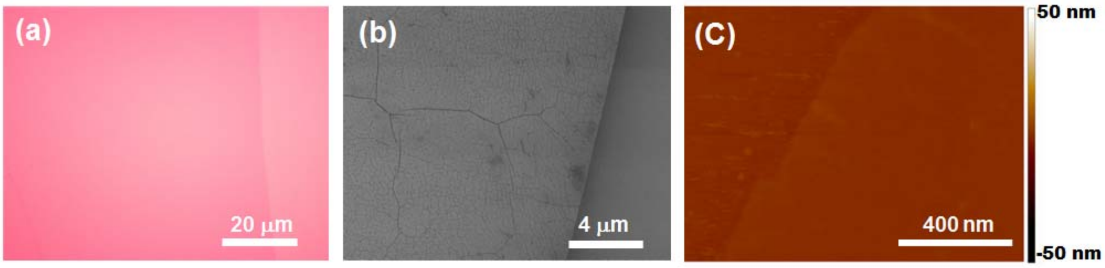

Supplementary Figure 5. **Characterization of the edge of the transferred graphene film.** (a) OM, (b) SEM and (c) AFM images of a rosin-transferred graphene film on a SiO_2/Si substrate, showing no rosin residue even at the edge with strong absorption ability.

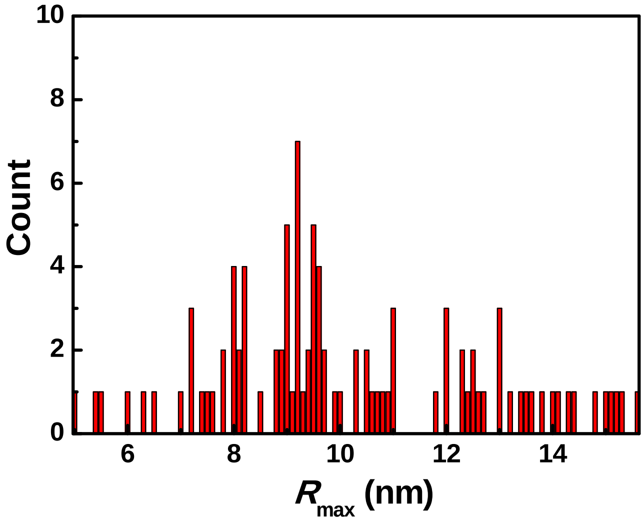

Supplementary Figure 6. Histogram of R_{max} collected from 100 randomly selected areas (5 × 5 μm^2) of a rosin-transferred graphene film on a SiO_2/Si substrate.

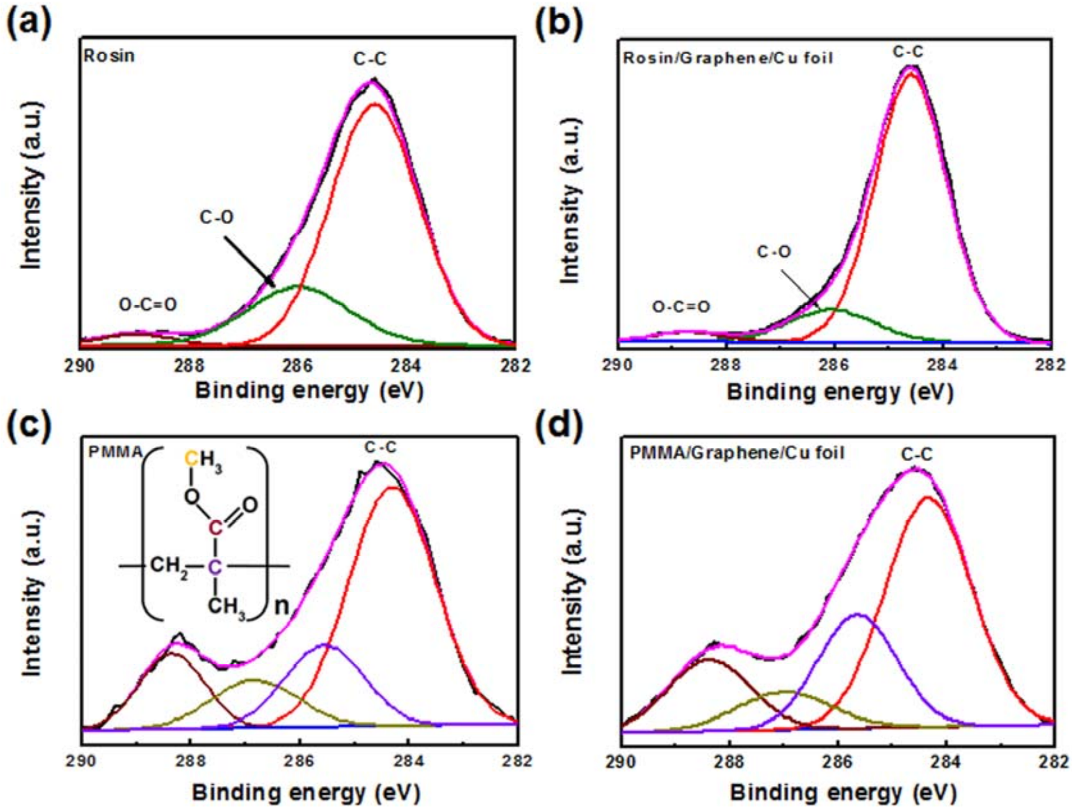

Supplementary Figure 7. XPS characterization of the graphene transferred with different support layers. High resolution C1s XPS spectra of (a) rosin, (b) rosin/Graphene/Cu foil, (c) PMMA, and (d) PMMA/Graphene/Cu foil. The wine (~288.8 eV), yellow (~286.8 eV), and violet (~285.6 eV) peaks in (c and d) respectively correspond to the carbon atoms in the carbonyl group, the methyl groups in the long ester side chain, and the carbon atoms of the short α-methyl side chain, as shown in the inset in (c).

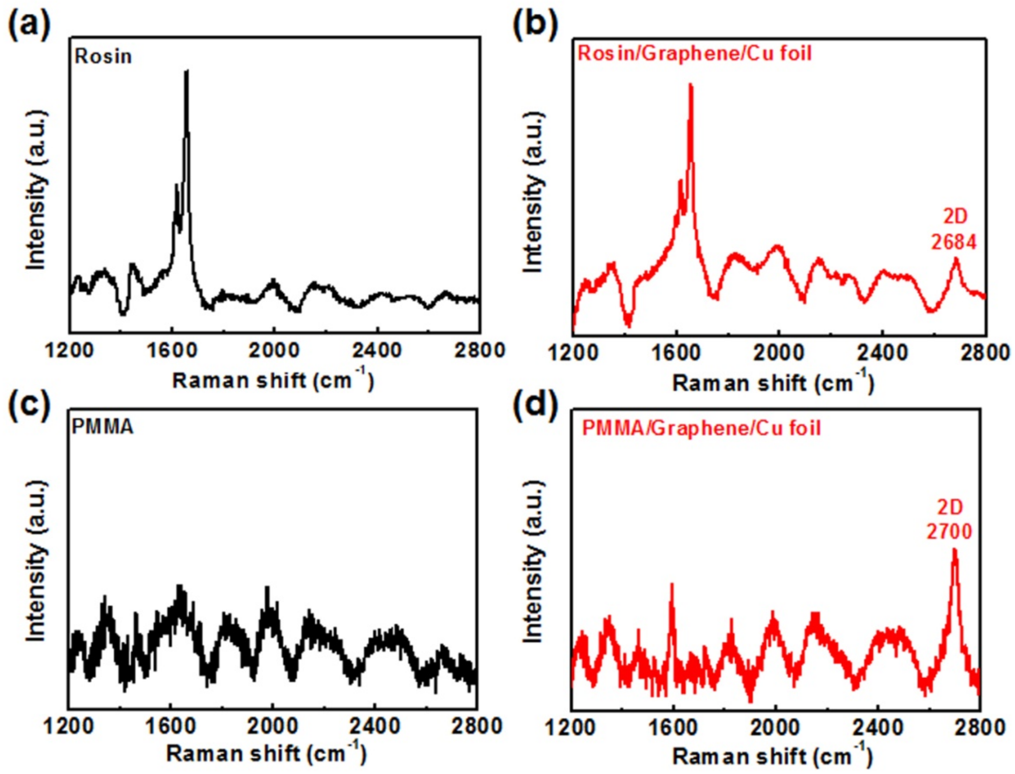

Supplementary Figure 8. **Raman spectroscopy characterization of the graphene transferred with different support layers.** Raman spectra of (a) rosin, (b) a rosin /graphene/Cu foil stack, (c) PMMA and (d) a PMMA/graphene/Cu foil stack.

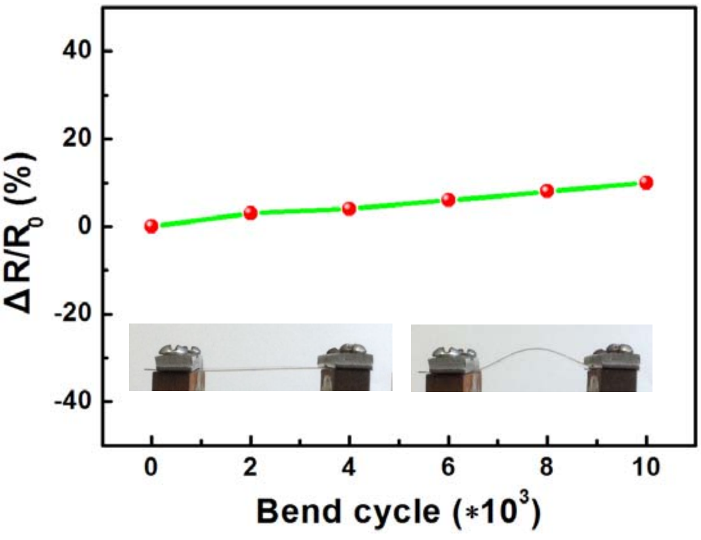

Supplementary Figure 9. Sheet resistance change of a monolayer graphene film on a PET substrate as a function of number of bending cycles. Insets are photographs of bending test.

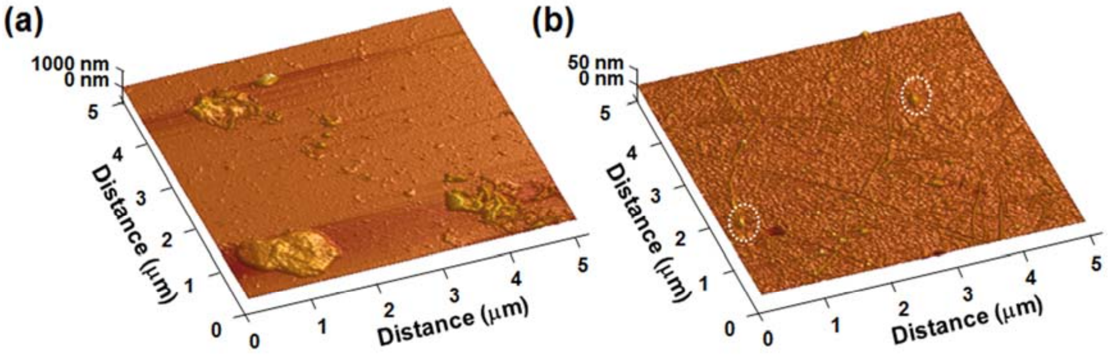

Supplementary Figure 10. Surface roughness characterization. 3D AFM images of (a) the PMMA- and (b) the rosin-transferred five-layer graphene films.

Supplementary Table 4. Performance comparison of CVD-grown graphene films transferred using different polymers as the support layer

Ref.	Graphene layers	Transfer method or polymer used	R_s (Ω per square)		T (%@550 nm)	RMS (nm)
			before doping	after doping (dopant)		
2	Monolayer	Sandwich (PMMA/SPPO1)	700-800	—	96.6	3.64
		PMMA	742	—	96.2	200[a]
3	Monolayer	PMMA	628	86 (TiOx and PEDOT:PSS)	>92@ 360-860 nm	0.976 (after modification)
8	Monolayer	PMMA	1500 ±200	90±10	96.4	—
10	Monolayer	PMMA	720	510 (CYTOP)	—	—
	Multilayer (4L)		330	200 (CYTOP)	> 85	—
11	Monolayer	Electrostatic force	255	—	—	—
	Multilayer (4L)		65	50 (HNO_3)	~ 90	
14	Monolayer	Dry transfer	150000	98000 (AgNW)	~ 97	—
				63000 (Au NP)	—	—
		Wet transfer	2000	990 (AgNW)	—	—

				400 (Au NP)	—	—
	Multilayer (8L)	Dry transfer	—	741 (AgNW)	~ 80	—
	Multilayer (12L)		—	110 (Au NP)	> 80	
35	Multilayer (1-3 nm in thickness)[b]	PMMA	230	—	72	~ 0.9
4	Bilayer[b]	PMMA	754.2	—	~95@520 ~ 800 nm	~ 1
13	Bilayer[b]	PMMA	265	~160 (PBASE)	~ 95.8	~ 1.8
7	Multilayer (4L)[c]	PMMA/Thermal release tape	87	~ 34 ($AuCl_3$) ~ 54 (HNO_3)	90	~ 3.4
1	Multilayer (~20L)[b]	Stamp method (PMMA)	310	—	~85 @522 nm	—
9	Multilayer (5-8L)[b]	—	< 200	—	80-85	3.3
46	Monolayer	PMMA	2200	—	—	—
		Polymer-free transfer	810	—	97.35	—
	Multilayer (4L)	PMMA	450	—	—	—
		Polymer-free transfer	230	—	89.4	—
47	Multilayer (6~10L)[a]	PDMS	~ 280	—	~ 80	—
48	Monolayer	PMMA	~ 125	—	97.4	—
		Thermal release tape	~272	~ 108 (HNO_3)	—	—[d]
	Multilayer (4L)	PMMA	~ 30	—	—	—
		Thermal release tape	~ 40	~ 30 (HNO_3)	~ 90	—
49	Monolayer	Self-adhesive film	~ 975	—	~ 96	~8.89 — 28
	Bilayer	Self-adhesive film	~ 888	—	~ 94.6	—
	Multilayer (3L)	Self-adhesive film	~ 750	—	~ 92	—
	Multilayer (4L)	Self-adhesive film	~ 712	—	~ 89.6	—
	Monolayer	First time reused self-adhesive film	~ 600	—	97.58	—
	Monolayer	Second time reused self-adhesive film	~ 1050	—	97.38	—
	Monolayer	Third time reused self-adhesive film	~ 1230	—	97.11	—
	Monolayer	Forth time reused self-adhesive film	~1080	—	97.23	—

Our work	Monolayer	Rosin	560	—	97.4	0.66
		PMMA	630	—	96.6	6.52
	Multilayer (5L)	Rosin	120	100 after oxidizing the top layer	85.1	3.51
		PMMA	200	165 after oxidizing the top layer	81.5	10.44

[a] The height of large PMMA residue particles, i.e., maximum roughness.

[b] Few-layer and multilayer graphene were obtained by direct CVD growth and then transferred to the target substrate.

[c] Multilayer graphene was obtained through layer-by-layer transfer, but only the first transferred graphene layer was coated with a polymer support layer during whole transfer process to avoid polymer residue between each layer.

[d] Although RMS was not measured, large particle-like adhesive residues can be clearly observed on the surface of the transferred graphene.

Supplementary References:

[1] Sun, T. *et al*. Multilayered graphene used as anode of organic light emitting devices. *Appl. Phys. Lett.* **96,** 133301(1-3) (2010).

[2] Han, Y. Y. *et al*. Clean surface transfer of graphene films *via* an effective sandwich method for organic light-emitting diode applications. *J. Mater. Chem. C* **2,** 201-207 (2014).

[3] Zhu, X. -Z. *et al*. The application of single-layer graphene modified with solution-processed TiOx and PEDOT:PSS as a transparent conductive anode in organic light-emitting diodes. *Org. Electron.* **14,** 3348-3354 (2013).

[4] Meng, H. *et al*. Bilayer graphene anode for small molecular organic

electroluminescence. *J. Phys. D: Appl. Phys.* **45,** 245103(1-6) (2012).

[5] Chang, H. X. *et al.* A transparent, flexible, low-temperature, and solution-processible graphene composite electrode. *Adv. Funct. Mater.* **20,** 2893-2902 (2010).

[6] Wu, J. B. *et al*. Organic light-emitting diodes on solution-processed graphene transparent electrodes. *ACS Nano* **4,** 43-48 (2010).

[7] Han, T. -H. *et al*. Extremely efficient flexible organic light-emitting diodes with modified graphene anode. *Nat. Photonics.* **6,** 105-110 (2012).

[8] Shin, S., Kim, J., Kim, Y. -H. & Kim, S. -Il. Enhanced performance of organic light-emitting diodes by using hybrid anodes composed of graphene and conducting polymer. *Curr. Appl. Phys.* **13,** S144-S147 (2013).

[9] Hwang, J. *et al*. Blue fluorescent organic light emitting diodes with multilayered graphene anode. *Mater. Res. Bull.* **47,** 2796-2799 (2012).

[10] Kwon, K. C., Kim, S., Kim, C., Lee, J. -L. & Kim, S. Y. Fluoropolymer-assisted graphene electrode for organic light-emitting diodes. *Org. Electron.* **15,** 3154-3161 (2014).

[11] Wang, D. -Y. *et al.* Clean-lifting transfer of large-area residual-free graphene films. *Adv. Mater.* **25,** 4521-4526 (2013).

[12] Wu, X. K. *et al*. Using a layer-by-layer assembly method to fabricate a uniform and conductive nitrogen-doped graphene anode for Indium-Tin Oxide-free organic light-emitting diodes. *ACS Appl. Mater. Interfaces* **6,** 15753-15759 (2014).

[13] Liu, Y. P. *et al*. “Quasi-freestanding” graphene-on-single walled carbon nanotube

electrode for applications in organic light-emitting diode. *Small* **10,** 944-949 (2014).

[14] Seo, J. -T. *et al*. Fully transparent quantum dot light-emitting diode integrated with graphene anode and cathode. *ACS Nano* **8,** 12476-12482 (2014).

[15] Matyba, P. *et al.* Graphene and mobile ions: the key to all-plastic, solution-processed light-emitting devices. *ACS Nano* **4,** 637-642 (2010).

[16] Chang, J. -H. *et al*. Solution-processed transparent blue organic light-emitting diodes with graphene as the top cathode. *Sci. Rep.* **5,** 9693(1-6) (2015).

[17] Meyer, J. *et al*. Metal oxide induced charge transfer doping and band alignment of graphene electrodes for efficient organic light emitting diodes. *Sci. Rep.* **4,** 5380(1-7) (2014).

[18] Meng, H. *et al*. Top-emission organic light-emitting diode with a novel copper/graphene composite anode. *Adv. Funct. Mater.* 23, 3324-3328 (2013).

[19] Hwang, J. *et al*. Multilayered graphene anode for blue phosphorescent organic light emitting diodes. *Appl. Phys. Lett.* **100,** 133304(1-4) (2012).

[20] Kuruvila, A. *et al*. Organic light emitting diodes with environmentally and thermally stable doped graphene electrodes. *J. Mater. Chem. C* **2,** 6940-6945 (2014).

[21] Li, F. S. *et al*. Fabrication of flexible conductive graphene/Ag/Al-doped zinc oxide multilayer films for application in flexible organic light-emitting diodes. *Org. Electron.* **14,** 2139-2143 (2013).

[22] Li, N. *et al*. Efficient and bright organic light-emitting diodes on single-layer graphene electrodes. *Nat. Commun.* **4,** 2294-2301 (2013).

[23] Jia, S. *et al*. Graphene oxide/graphene vertical heterostructure electrodes for

highly efficient and flexible organic light emitting diodes. *Nanoscale* **8,** 10714-10723 (2016).

[24] Han, T. -H. *et al*. Versatile p-type chemical doping to achieve ideal flexible graphene electrodes. *Angew. Chem. Int. Ed.* **55,** 1-6 (2016).

[25] Lee, J. *et al*. Synergetic electrode architecture for efficient graphene-based flexible organic light-emitting diodes. *Nat. Commun.* **7,** 11791 (1-9) (2016).

[26] Kim, S. *et al*. Challenge beyond graphene: metal oxide/graphene/metal oxide electrodes for optoelectronic devices. *ACS Appl. Mater. Interfaces* **8,** 12932-12939 (2016).

[27] Wang, Y., Chen, X. H., Zhong, Y. L., Zhu, F. R. & Loh, K. P. Large area, continuous, few-layered graphene as anodes in organic photovoltaic devices. *Appl. Phys. Lett.* **95,** 063302(1-3) (2009).

[28] Park, H., Rowehl, J. A., Kim, K. K., Bulovic, V. & Kong, J. Doped graphene electrodes for organic solar cells. *Nanotechnology* **21,** 505204(1-6) (2010).

[29] Tung, V. C. *et al*. Low-temperature solution processing of graphene-carbon nanotube hybrid materials for high-performance transparent conductors. *Nano Lett.* **9,** 1949-1955 (2009).

[30] Wang, Y., Tong, S. W., Xu, X. F., Özyilmaz, B. & Loh, K. P. Interface engineering of layer-by-layer stacked graphene anodes for high-performance organic solar cells. *Adv. Mater.* **23,** 1514-1518 (2011).

[31] Xu, Y. F. *et al*. Polymer photovoltaic devices with transparent graphene electrodes produced by spin-casting. *Carbon* **48,** 3293-3311 (2010).

[32] Yin, Z. Y. *et al*. Organic photovoltaic devices using highly flexible reduced graphene oxide films as transparent electrodes. *ACS Nano* **4,** 5263-5268 (2010).

[33] Geng, J. X. *et al*. A simple approach for preparing transparent conductive graphene films using the controlled chemical reduction of exfoliated graphene oxide in an aqueous suspension. *J. Phys. Chem. C* **114,** 14433-14440 (2010).

[34] Kymakis, E., Savva, K., Stylianakis, M. M., Fotakis, C. & Stratakis, E. Flexible organic photovoltaic cells with in situ nonthermal photoreduction of spin-coated graphene oxide electrodes. *Adv. Funct. Mater.* **23,** 2742-2749 (2013).

[35] Arco, L. G. D. *et al*. Continuous, highly flexible, and transparent graphene films by chemical vapor deposition for organic photovoltaics. *ACS Nano* **4,** 2865-2873 (2010).

[36] Jo, G. *et al*. Tuning of a graphene-electrode work function to enhance the efficiency of organic bulk heterojunction photovoltaic cells with an inverted structure. *Appl. Phys. Lett.* **97,** 213301(1-3) (2010).

[37] Lee, D. H. *et al*. Highly stable and flexible silver nanowire–graphene hybrid transparent conducting electrodes for emerging optoelectronic devices. *Nanoscale* **5,** 7750-7755 (2013).

[38] Lee, Y. -Y. *et al*. Top laminated graphene electrode in a semitransparent polymer solar cell by simultaneous thermal annealing/releasing method. *ACS Nano* **5,** 6564-6570 (2011).

[39] Tong, S. W., Wang, Y., Zheng, Y., Ng, M. -F. & Loh, K. P. Graphene intermediate layer in tandem organic photovoltaic cells. *Adv. Funct. Mater.* **21,** 4430-4435 (2011).

[40] Park, H., Chang, S., Smith, M., Gradečak, S. & Kong, J. Interface engineering of graphene for universal applications as both anode and cathode in organic photovoltaics. *Sci. Rep.* **3,** 1581(1-8) (2013).

[41] Moaven, S., Naji, L., Taromi, F. A. & Sharif, F. Effect of bending deformation on photovoltaic performance of flexible graphene/Ag electrode based polymer solar cells. *RSC Adv.* **5,** 30889-30901 (2015).

[42] Konios, D. *et al*. Reduced graphene oxide micromesh electrodes for large area, flexible, organic photovoltaic devices. *Adv. Funct. Mater.* **25,** 2213(1-9) (2015).

[43] Lin, Y. -C. *et al*. Graphene annealing: how clean can it be? *Nano Lett.* **12,** 414-419 (2012).

[44] Park, H., Brown, P. R., Bulović, V. & Kong, J. Graphene as transparent conducting electrodes in organic photovoltaics: studies in graphene morphology, hole transporting layers, and counter electrodes. *Nano Lett.* **12,** 133-140 (2012).

[45] Kim, H. H. *et al*. Clean transfer of wafer-scale graphene *via* liquid phase removal of polycyclic aromatic hydrocarbons. *ACS Nano* **9,** 4726-4733 (2015).

[46] Lin, W. -H. *et al*. A direct and polymer-free method for transferring graphene grown by chemical vapor deposition to any substrate. *ACS Nano* **8,** 1784-1791 **(**2014).

[47] Kim, K. S. *et al*. Large-scale pattern growth of graphene films for stretchable transparent electrodes. *Nature* **457,** 706-710 (2009).

[48] Bae, S. *et al.* Roll-to-roll production of 30-inch graphene films for transparent electrodes. *Nat. Nanotechnol.* **5**, 574-578 (2010).

[49] Hong, B. H., Kim, S. J., Park, M. J. & Jo, I. S. Transferring method of graphene

using self-adhesive film. (US 20150314579 A1 / WO 2014109619 A1) Nov. 5, (2015).
